# Text messaging as a tool to improve cancer screening programs (M-TICS Study): A randomized controlled trial protocol

**DOI:** 10.1371/journal.pone.0245806

**Published:** 2021-01-22

**Authors:** Nuria Vives, Albert Farre, Gemma Ibáñez-Sanz, Carmen Vidal, Gemma Binefa, Núria Milà, Maria Jose Pérez-Lacasta, Noemie Travier, Llucia Benito, Josep Alfons Espinàs, Guillermo Bagaria, Montse Garcia

**Affiliations:** 1 Cancer Screening Unit, Catalan Institute of Oncology, Hospitalet de Llobregat, Barcelona, Spain; 2 Early Detection of Cancer Research Group, EPIBELL Program, Bellvitge Biomedical Research Institute, L’Hospitalet de Llobregat, Barcelona, Spain; 3 Consortium for Biomedical Research in Epidemiology and Public Health (CIBEResp), Madrid, Spain; 4 School of Health Sciences, University of Dundee, Dundee, United Kingdom; 5 Oncology Data Analytics Program, Catalan Institute of Oncology, Hospitalet de Llobregat, Barcelona, Spain; 6 Gastroenterology Department, Bellvitge University Hospital, L'Hospitalet de Llobregat, Barcelona, Spain; 7 Colorectal Cancer Group, ONCOBELL Program, Bellvitge Biomedical Research Institute, L’Hospitalet de Llobregat, Barcelona, Spain; 8 Department of Economics, University Rovira i Virgili, Reus, Spain; 9 Research Group on Economic Evaluation and Health (GRAES), Reus, Spain; 10 Fundamental Care and Medical Surgical Nursing Department, School of Nursing, University of Barcelona, L’Hospitalet de Llobregat, Barcelona, Spain; 11 Catalonian Cancer Strategy, Department of Health, Government of Catalonia, Barcelona, Spain; 12 Official College of Pharmacists of Barcelona, Barcelona, Spain; Leibniz Institute for Prevention Research and Epidemiology BIPS, GERMANY

## Abstract

**Background:**

Short message service (SMS) based interventions are widely used in healthcare and have shown promising results to improve cancer screening programs. However, more research is still needed to implement SMS in the screening process. We present a study protocol to assess the impact on health and economics of three targeted SMS-based interventions in population-based cancer screening programs.

**Methods/Design:**

The M-TICs study is a randomized controlled trial with a formal process evaluation. Participants aged 50–69 years identified as eligible from the colorectal cancer (CRC) and breast cancer (BC) screening program of the Catalan Institute of Oncology (Catalonia, Spain) will be randomly assigned to receive standard invitation procedure (control group) or SMS-based intervention to promote participation. Two interventions will be conducted in the CRC screening program: 1) Screening invitation reminder: Those who do not participate in the CRC screening within 6 weeks of invite will receive a reminder (SMS or letter); 2) Reminder to complete and return fecal immunochemical test (FIT) kit: SMS reminder versus no intervention to individuals who have picked up a FIT kit at the pharmacy and they have not returned it after 14 days. The third intervention will be performed in the BC screening program. Women who had been screened previously will receive an SMS invitation or a letter invitation to participate in the screening.

As a primary objective we will assess the impact on participation for each intervention. The secondary objectives will be to analyze the cost-effectiveness of the interventions and to assess participants’ perceptions.

**Expected results:**

The results from this randomized controlled trial will provide important empirical evidence for the use of mobile phone technology as a tool for improving population-based cancer screening programs. These results may influence the cancer screening invitation procedure in future routine practice.

**Trial registration:**

Registry: NCT04343950 (04/09/2020); clinicaltrials.gov.

## Introduction

Cancer screening programs base their potential benefit on identifying a specific disease in an asymptomatic phase to reduce its burden by using evidence-based, feasible and efficient screening strategies. For this purpose, a test or examination is used to identify healthy individuals who have a higher probability of having the condition of interest, so that an early treatment or intervention can be offered [1,2]. The effectiveness of an organized cancer screening program depends on the quality of each part of the process involving invitation, testing and diagnosis, as well as planning, monitoring and evaluation. A major determinant of the success of a cancer screening program is the participation of the target population. Equity of access to screening is still a challenge in achieving high participation rates [3,4]. In Catalonia, BC and CRC population-based screening programs are offered free of charge. However, participation rates in both screening programs remain below the desirable participation rates of 70% and 65% respectively, as recommended by the European Guidelines for Quality Assurance in BC and CRC Screening [5,6]. Moreover, in CRC, we have identified a non-negligible percentage of individuals who collect the fecal immunochemical test (FIT) at the community pharmacy but do not return it, which presents another opportunity for a targeted intervention [7].

Currently, eligible population are invited to participate in cancer screening programs by postal letter [6,8,9]; however mobile phone technology is emerging as a powerful tool in health care systems [10–12]. In particular, short message service (SMS) is now widely used by health care systems to support a broad range of health behaviors [13]. SMS provides a low-cost, accessible and scalable mode of intervention delivery, allowing almost instantaneous communication without relying on smart phone ownership, internet access or high degrees of digital literacy [14]. A systematic review of SMS reminder interventions showed a moderate increase in BC screening appointments (4.5% to 15%) and a smaller participation improvement in CRC screening (0.6% to 3.3%) [15]. Regarding CRC screening, a randomized controlled trial in a CRC screening program did not observe an overall increase in participation, but sending an SMS reminder increased the number of first-time participants [16]. Two other studies reported an increased participation in the CRC screening program by sending SMS reminders to lower socioeconomic status individuals [17,18]. Finally, a quasi-experimental study in Catalonia showed the acceptability of SMS reminders in the BC screening program [19].

### Objectives

- To assess the effectiveness of SMS-based interventions to increase participation in the current CRC and BC screening programs of the metropolitan area of Barcelona.
- To analyze the cost-effectiveness of adding SMS-based interventions to CRC and BC screening programs within one year.
- To assess participants’ perceptions and experiences of SMS-based interventions implemented across the two screening programs.

## Methods

The underlying protocol follows SPIRIT Statement: Defining standard protocol items for clinical trials [20].

### Design

The M-TICS study is a prospective, randomized-controlled, open labeled study registered on clinicaltrials.gov, April 9, 2020 (NCT04343950) [21] (S1 Table).

### Setting and eligibility criteria

The two population-based cancer screening programs included in this study are both managed by the Catalan Institute of Oncology (Catalonia, Spain).

The BC screening program offers biennial screening mammography to a target population of 160,000 women aged 50–69 years from the Southern metropolitan area of Barcelona. About 80,000 women are invited each year, half of whom have participated in the previous round. Women are excluded according to the following criteria: personal history of breast cancer; bilateral prophylactic mastectomy, female-to-male gender reassignment, terminal illness and, severe disabling condition.

The CRC screening program has a target population of 495,000 women and men aged 50–69 years from Northern and Southern metropolitan areas of Barcelona. Subjects are excluded according to the following criteria: gastrointestinal symptoms, history of personal CRC or adenomas, high-risk familial history or hereditary CRC, inflammatory bowel disease, colonoscopy in the previous five years or a fecal occult hemoglobin test within the last two years, terminal disease and, severe disabling conditions.

### Screening invitation process

#### Colorectal cancer (standard procedure)

An invitation letter, where participants are asked to pick up a FIT kit at any nearby community pharmacy participating in the CRC program, is sent accompanied by an informative leaflet on CRC. Invitations are sent according to Primary healthcare area of residence. On the sixth week, a reminder invitation letter to non-respondents is sent. Community pharmacies collect completed FIT kits and send them to reference laboratory to be processed. Participants with a negative FIT result receive a recommendation for biennial screening while individuals with a positive FIT result are contacted by both postal mail and telephone and offered a diagnostic colonoscopy.

#### Breast cancer (standard procedure)

An invitation letter with a scheduled appointment for a mammography accompanied by an informative leaflet on BC is sent to the target population one month before the appointment. The appointment does not need to be confirmed but can be rescheduled or cancelled if necessary. Invitations are sent by individuals' date of birth. An SMS reminder is sent 3 days before the appointment to all women with a mobile phone number registered in the Information System for Monitoring BC Screening. Participants with a negative mammography receive a recommendation for biennial screening while individuals with an abnormal result are offered a diagnostic follow up.

### Intervention

**Fig 1:**
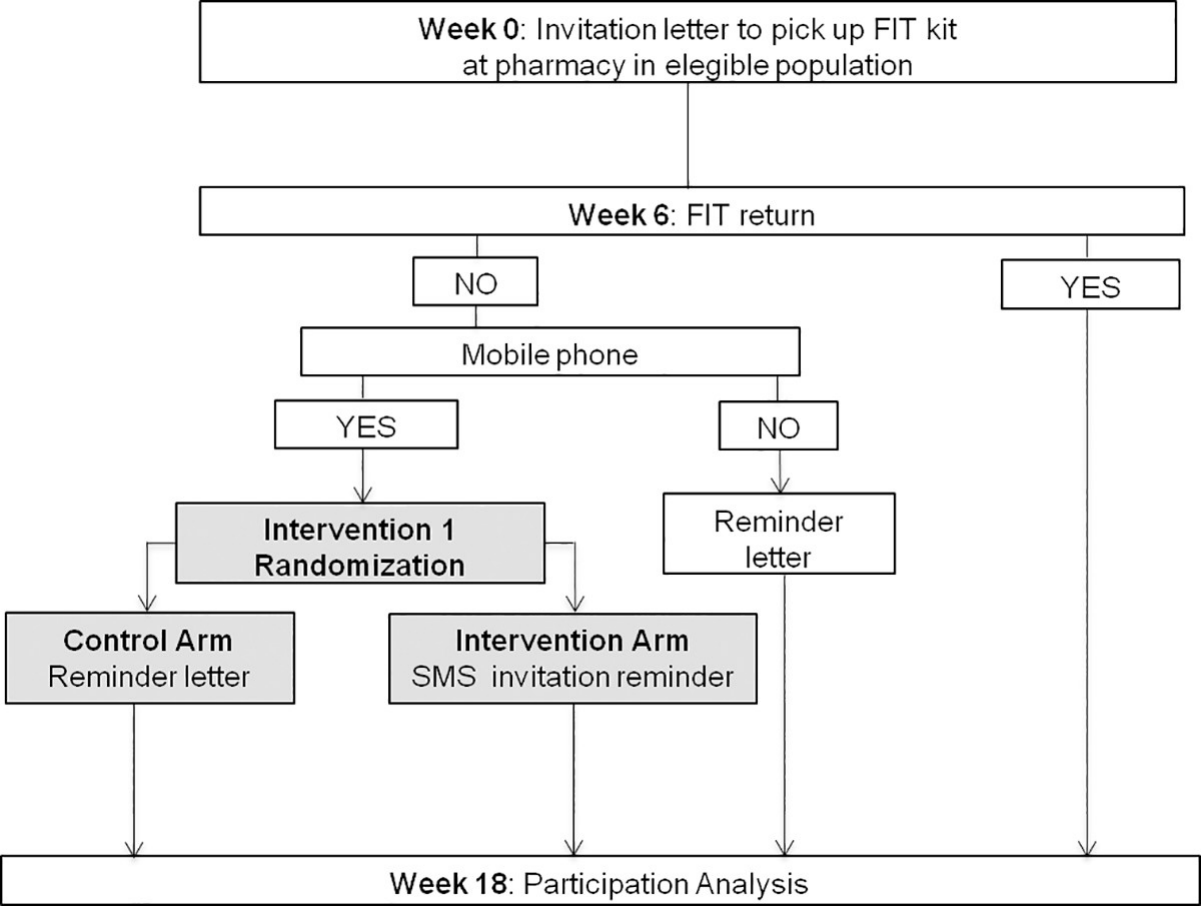
CONSORT diagram of screening invitation reminder of colorectal cancer screening.

**Fig 2:**
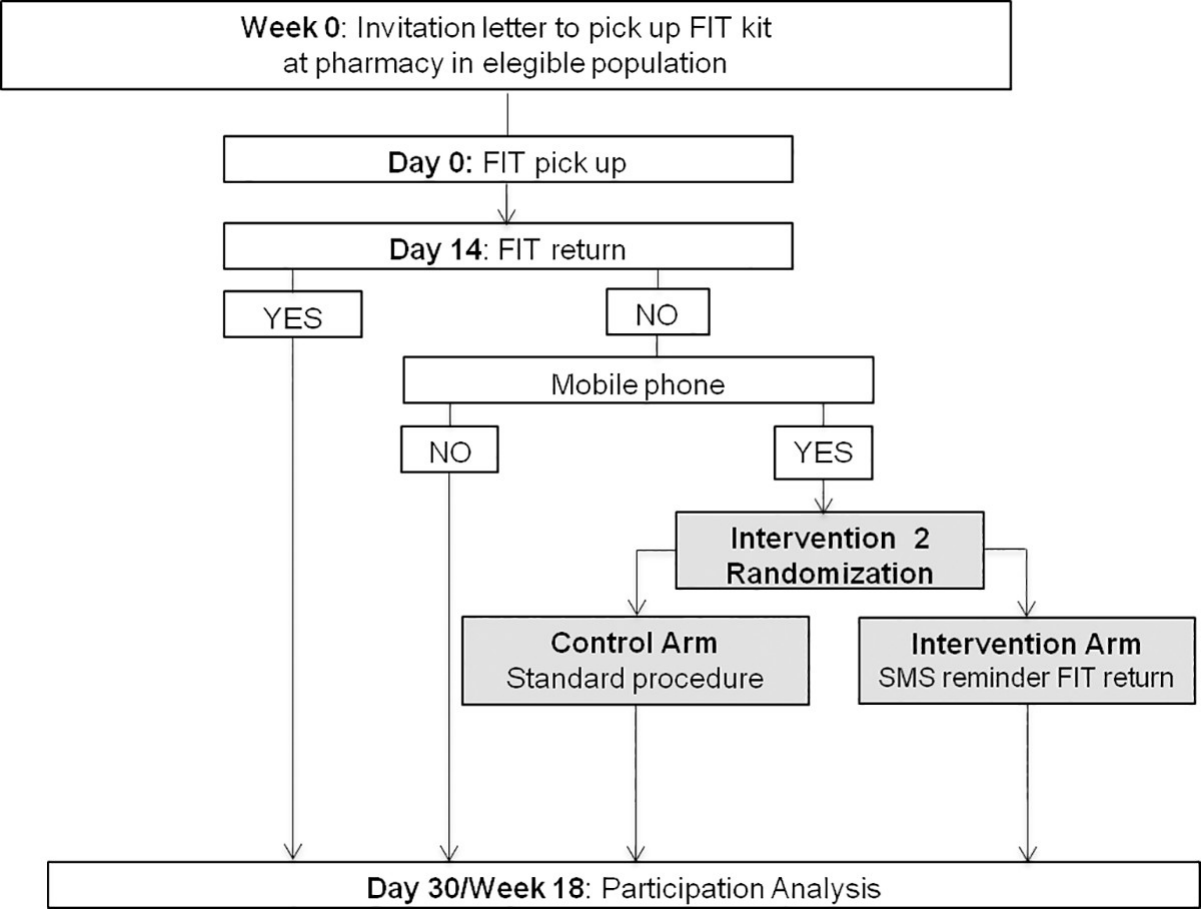
CONSORT diagram of reminder to complete and return FIT kit for colorectal cancer screening.

**Fig 3:**
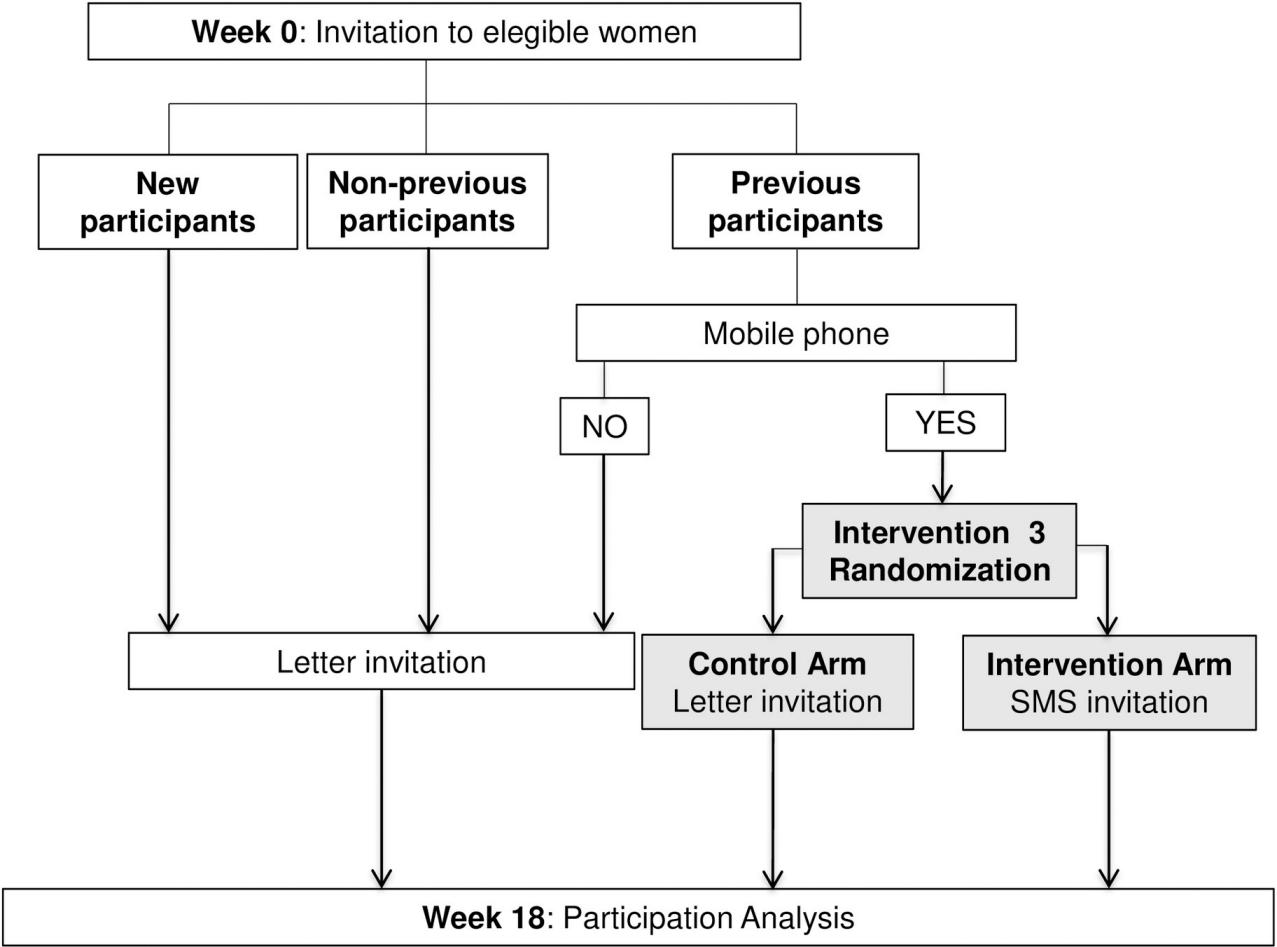
CONSORT diagram of screening invitation for successive participants for breast cancer screening.

Figs 1–3 shows the CONSORT diagram of the M-TICS Study on each of the three SMS-based interventions. The SMS will include the name of the responsible of the CRC screening (Catalan Institute of Oncology), the purpose of the message, and guidance on where to get more information.

#### Intervention 1: Screening invitation reminder for CRC screening

*Setting*: Primary healthcare area s of the metropolitan area of Barcelona whose target population will be invited to CRC Screening.

*Participants*: Men and women between 50 and 69 years old who have not completed the FIT kit within 6 weeks of being invited to CRC screening.

*Interventions*: Individuals without a mobile phone registered will receive the standard postal reminder letter procedure. Individuals with a mobile phone registered will be randomly assigned to either receive the standard postal reminder letter or the SMS reminder if they have not completed the FIT kit 6 weeks into their screening episode (Fig 1).

*Sample size*: Sample size has been calculated to detect differences in participation among intervention and control groups stratifying by screening behavior (first-time invitees, non-participants and previous participants). We have estimated that 70% of invitees will need a reminder at 6 weeks, 15% of individuals will not have a mobile phone registered and, 10% of phone numbers will be wrongly recorded. To detect a difference of 3% in participation between the intervention and the control groups for each stratum separately and considering a two-sided alpha of 5% and a power of 90%, we will require a total of 25,572 individuals.

#### Intervention 2: Reminder to complete and return FIT kit for CRC screening

*Setting*: Primary healthcare area s of the metropolitan area of Barcelona whose target population will be invited to CRC Screening.

*Participants*: Men and women between 50 and 69 years of age invited to CRC screening.

*Interventions*: Individuals with a registered mobile phone will be randomly assigned to either receive the standard procedure (no FIT kit return reminder) or an SMS FIT kit return reminder if the FIT kit has not been returned at the pharmacy at 14 days from pick-up, the point at which 90% of the people return the completed FIT kit (Fig 2).

*Sample size*: Calculations were made to detect differences in participation among intervention and control groups. We have estimated that annually about 14,000 individuals will not complete the FIT kit in 14 days since pick-up at pharmacy, 15% of individuals will not have a mobile phone registered and, 10% of phone numbers will be wrongly recorded. Using these estimates, to detect a difference of 3% in participation between the intervention and the control groups and considering a two-sided alpha of 5% and a power of 90%, a total of 10,174 individuals we will require (5,087 individuals in each group).

#### Intervention 3: Screening invitation for successive participants in BC screening

*Setting*: The target population from the Southern metropolitan area of Barcelona who will be invited to BC screening.

*Participants*: Women between 50 and 69 years of age invited to BC screening with previous screening behavior.

*Interventions*: Women without a mobile phone registered will receive the standard postal invitation letter procedure. Women aged 52–69 with a mobile phone registered who have participated in the BC screening program in the immediately preceding round will be randomly assigned to either receive the standard postal invitation letter procedure or an SMS-only invitation (Fig 3).

*Sample size*: Calculations were made to detect a participation difference of 2.0 percent points between the control and this intervention group (86% vs 84%), respectively. We estimated that about 80,000 women are invited each year, 54,7% of whom participated in the previous round, 15% of individuals will not have a mobile phone registered and, 10% of phone numbers will be wrongly recorded. Participation among women with a regular successive screening is 86%. Using these estimates, a type 1 error rate of 5%, and a power of 90% in a one-side test, a target sample size of 10, 908 individuals will be needed.

### Assignment of intervention

#### Randomization and allocation

Assignment of interventions 1 and 2 has been modified from the original protocol. A cluster randomization was intended, considering as a cluster “week of screening invitation”. A pilot study was conducted and a selection bias in intervention assignment was observed. Therefore, after verifying its technological feasibility, a simple randomization of individuals will be performed.

A list of eligible individuals will be generated on the basis of the inclusion and exclusion criteria for each one of the three interventions. In each intervention, simple randomization will be performed to allocate the participants to the standard procedure or the intervention. Individuals will be randomized in a 1:1 ratio to either the intervention or control. The random allocation sequences will be obtained using a computer-generated random number to derive allocation sequence prior to enrollment in the study. Random number table will be kept in a secure database set that will only accessible by authorized researchers.

#### Blinding

It will not be possible to blind any of interventions implemented to the participants, the study investigators or data analysts. However, individuals registered at the Information System for Monitoring CRC Screening and BC Screening will not be informed that they are participants in a research study.

### Outcome measures

The primary outcome measure for all three interventions will be participation as a dichotomous variable (no/yes) in all randomized participants (intention-to-treat analysis):

- For interventions 1 and 2 (CRC screening) participation will be defined as individuals returning an adequate FIT kit within 18 weeks of the invitation. In the intervention 2 we will also analyze immediate participation within 30 days of FIT kit pick up.
- For intervention 3 (BC screening) participation will be defined as women with a performed mammography in those randomized within 12 weeks of the invitation.

An additional secondary outcome measure will be cost-effectiveness. Effectiveness will be measured through the number of people who have participated in the screening and, if applicable, the proportion of advanced malignancies detected by increased participation will be calculated. The result will be specified as an incremental ratio between costs and effectiveness (ICER) this is defined as cost per participant.

#### Process evaluation

All three interventions will include an embedded process evaluation including: a participants survey (n = 100 for each intervention) to test reading and understanding of text messages among trial participants; and qualitative semi-structured interviews with trial participants’ to explore their experiences of the interventions. We will use a purposeful, maximum variation sampling strategy. Sample size will be determined by data saturation (with an estimated sample of n = 36 for interventions 1 and 2, and n = 24 for intervention 3).

### Data collection and management

Baseline characteristics that might affect whether participants get screened will be included directly from the Information System for Monitoring CRC or BC Screening: sex, age, primary healthcare area of reference, previous screening behavior (first-time invited, non-participants, and participants in the previous round), number of FIT per person, invitation date, date of completion test (FIT or mammography), and screening results. Since no individual information on socioeconomic status is available, a socioeconomic index calculated for Primary healthcare area of the Catalan territory will be used. This index uses aggregated indicators of income, employment, health and disability and education to generate a scale from 0 (least deprived) to 100 (most deprived) [22]. Finally, a variable on accessibility to postal mail will be calculated based on the population growth in the last period and population density of assigned primary healthcare area s (easy access areas/hard to reach areas) [19].

Once each participant’s data set is completed, it is de-identified, entered into a central database, and maintained securely by the principal investigator. All data and relevant correspondence will be stored according to current legislation on the protection of personal data and archived at trial sites for a minimum of 5 years.

### Statistical analysis

#### Primary effectiveness analysis

Socio-demographic characteristics of the study population for each of the three interventions will be compared between control and intervention group to assess any imbalances in covariates during randomization. Continuous variables will be analyzed using Student's t test, and categorical variables using Chi-square tests. The primary outcome as the proportion who participated in each group for the three interventions will be analyzed using bivariate logistic regression models, and a multivariate logistic regression analysis with stepwise forward variable selection method will be used. The variables with P-values < 0.10 in the simple regression analysis will be included in the final multiple models. For intervention 2, we will compare change in participation at 18 weeks from that at 30 days between control and intervention groups. Results will be presented using odds ratios (ORs), 95% confidence intervals (95% CIs), and P-values <0.05 will be considered as statistically significant.

In addition, for intervention 3, two months interim analysis is planned into the trial and it will be stopped if the participation in the intervention group (SMS-only invitation) is reduced by 3 percent points or more compared to standard invitation (letter).

#### Secondary cost-effectiveness analysis

The cost-effectiveness analysis will be performed through a decision tree for each of three SMS text message interventions, taking into account the use of SMS on costs and results. Costs will be analyzed from the healthcare funder perspective for each population-based screening program, including: a) Labor costs, consumables and depreciation of property, facilities and equipment, as well as "overheads". The SMS alternative will specifically include the personnel and infrastructure fixed costs; b) Diagnosis costs, specifically the costs of evaluating cases with a positive screening FIT. Future costs will not be included. The temporary period of analysis will be 1 year. A deterministic sensibility analysis will be applied to test the robustness of the results.

#### Process evaluation

Participant survey data will be analyzed using descriptive statistics. Qualitative data from individual interviews will be analyzed using a thematic approach. Data across interventions and across the quantitative and qualitative arms of the process evaluation will be brought together into an integrated mixed-methods analysis.

### Timeline

See Table 1 for an overview of the study timeline.

**Table 1:** M-TICS timeline.

		STUDY PERIOD					
		Eligibility	Enrollment & Allocation	Post-allocation			Close-out
	**TIMEPOINT**	***-t***_***1***_	***0***	***t***_***1***_	***t***_***2***_	***t***_***3***_	***t***_***4***_
	**Enrollment**						
**ENROLLMENT**	**Eligibility screen**						
	**Random allocation**						
**INTERVENTIONS**	***Intervention 1***						
	***Intervention 2***						
	***Intervention 3***						
**ASSESSMENTS**	***Baseline variables***						
	***Outcome variables***						
	***Process evaluation***						

The recruitment process of the study will take place during 2021. For intervention 1, individuals invited to CRC screening will be enrolled into study on the sixth week and randomize to stay until the screening episode is closed for adequate FIT kit at 18 week. For intervention 2, individuals invited to CRC screening during 2021 will be enrolled into study on the fourteenth day from FIT kit pick up and randomize to stay until the screening episode is closed for adequate FIT kit at 18 week. Finally, for intervention 3 women with previous screening behavior invited during 2021 will be enrolled into study at day 0 and randomize to stay until the screening episode is closed for adequate mammography at 12 week.

### Data monitoring

Investigators from the Cancer Screening Unit at the Catalan Institute of Oncology will be involved in the design and the implementation of the interventions. The principal investigator will be responsible for supervising the administrative management of the study and the execution of the trial, record keeping and data management. This study will assess three targeted SMS-based interventions that are highly unlikely to be related to serious adverse events. Thus, no data monitoring committee or auditing will be needed. However, the principal investigator will take care of monitoring and will be responsible for biweekly reports on the progress of the study.

## Ethics and dissemination

The M-TICS study was approved by the Ethics Committee of the Bellvitge University Hospital (approval numbers PR042/20), who deemed that informed consent of the participants was not needed because the study will be embedded in a routine screening service. The study is performed in accordance with Good Clinical Practice and the Declaration of Helsinki. BC and CRC screening program follow the regulations on general public health and data protection [23–25]. Both screening programs accomplish specific protocols based on the existing guidelines [26]. Data underlying reported findings will be deposited in the Universitat de Barcelona Digital Repository (http://diposit.ub.edu/dspace/) to be publicly available.

The results of the trial will be published in peer-reviewed journals and presented in conferences. Authorship credit will be based on substantial contribution to conception and design, execution, or analysis and interpretation of data. All authors will be involved in drafting the article or revising it critically for important intellectual content and final approval of the manuscript.

### Potential amendments

The recruitment of participants is scheduled to be carried out during 2021. Nevertheless, cancer screening programs might be temporally suspended as a response to controlling the COVID-19 pandemic and that would affect the project timeline. In case a need for modification should arise, it will be registered and reported in this journal.

## Expected results

The spread of digital technologies is driving a transformation of the economy and society towards a digital media. Naturally, the transition to a digital society model can only be achieved when the majority of individuals habitually use mobile telephones, the Internet, and other services to profit from these new opportunities. Innovations in screening programs are necessary to optimize economic resources.

We expect a small effect in the overall participation in the SMS-based intervention tested in both BC and CRC screening programs. Since participation in BC screening is higher among women with previous screening, the margin for improvement is limited. However, in CRC screening we expect to find higher differences in participation for certain subgroups, such as first time invitees and the youngest age groups. We expect that the implementation of such interventions will improve effectiveness of screening since there is a high rate of continued participation in our programs (>80%) [18].

SMS would allow improving accessibility to screening in individuals with a high geographic mobility, which may be excluded from the screening programs due to frequent changes of address and those with difficult access to postal mail.

The targeted SMS-based intervention to those population subgroups with greater motivation to participate in the programs, such as those individuals who pick up the FIT kit at the pharmacy, are more likely to be successful than strategies addressing to all non-participants. In addition, it would also allow us to keep costs low.

Furthermore, time to FIT completion can also be shortened and the proportion of participants requiring more than one FIT can be reduced.

The findings from the process evaluation will be useful in interpreting the results of the study and identifying strategies to refine and optimize the tested interventions.

In the long term, the study may provide important empirical evidence for the use of mobile technology as a tool for the invitation procedure of population-based cancer screening programs and the effect on participation in these screenings. In future, these results may influence the invitation procedure to cancer screening in routine practice.

## Supporting information

S1 Checklist Recommended items to address in a clinical trial protocol and related documents*. (DOC)

S1 Table World Health Organization trial registration data set. (DOCX)

S1 File (DOCX)

S2 File (PDF)
