# Supplementary material for: Text messaging as a tool to improve cancer screening programs (M-TICS Study): A randomized controlled trial protocol
Source: PLoS One. 2021 Jan 22;16(1):e0245806. doi: 10.1371/journal.pone.0245806 (PMC7822525; doi:10.1371/journal.pone.0245806)
Supplement: S1 Table — (DOCX) [file pone.0245806.s002.docx]

**Supplementary Table 1. World Health Organization Trial Registration Data Set.**

| **Data category** | **Information** |
| --- | --- |
| Primary registry and trial identifying number | ClinicalTrials.gov NCT04343950 |
| Date of registration in primary registry | April 9, 2020 |
| Secondary identifying numbers | PI19/00226 |
| Source(s) of monetary or material support | The National Institute of Health Carlos III (ISCIII) |
| Primary sponsor | The National Institute of Health Carlos III (ISCIII) |
| Secondary sponsor(s) | Department of Universities and Research of the Government of Catalonia |
| Contact for public queries | *Cancer Screening Unit*, +34932607959, [prevenciocolon@iconcologia.net](mailto:prevenciocolon@iconcologia.net)  ] |
| Contact for scientific queries | *Montse Garcia*, PhD, +34932607205, [mgarcia@iconcologia.net](mailto:mgarcia@iconcologia.net), Cancer Screening Unit, Catalan Institute of Oncology Catalonia, Spain |
| Public title | SMS Messaging as a Tool to Improve Cancer Screening Programs (M-TICS) |
| Scientific title | Implementation of Text Messaging (SMS) as an Improvement Tool in Population-based Cancer Screening Programs |
| Countries of recruitment | Spain |
| Health condition(s) or problem(s) studied | Mass Screening, Colorectal Cancer, Breast Cancer |
| Intervention(s) | Active comparator: SMS interventions: 1) SMS reminder invitation at colorectal cancer screening; 2) SMS reminder at return colorectal cancer screening test; 3) Breast cancer screening SMS invitation. Placebo comparator: Usual Care: 1) Reminder invitation letter at colorectal cancer screening; 2) No intervention; 3) Breast cancer screening letter invitation. |
| Key inclusion and exclusion criteria | Inclusion Criteria: Men and women 50-69 years of aged invited to colorectal cancer screening (intervention 1 and 2); Women 50-69 years of aged invited to breast cancer screening (intervention 3); Registered at the Catalan Healthcare Database.  Exclusion Criteria: Individuals without a mobile phone number registered at the Catalan Healthcare Database. |
| Study type | Interventional Allocation: randomized Intervention model: parallel assignment Masking: Open Label ( Primary purpose: Health Services Research |
| Date of first enrolment | January 2021 |
| Target sample size | 20 000 (anticipated) |
| Recruitment status | Not yet recruiting |
| Primary outcome(s) | Intervention 1: Participation in colorectal cancer screening program 18 weeks after sending the screening invitation [Time Frame: 18 weeks]  Intervention 2: Participation in colorectal cancer screening program among individuals who pick up the test at the pharmacy 18 weeks after sending the screening invitation [Time Frame: 18 weeks]  Intervention 3: Participation in breast cancer screening program 8 weeks after sending the screening invitation letter [Time Frame: 8 weeks] |
| Key secondary outcomes | Incremental cost ratio: A cost-effectiveness analysis of the three interventions will be carried out. The incremental cost ratio of the interventions between cost variation and effectiveness variation will be calculated. [Time Frame: 18 weeks] |
