# Supplementary material for: Text messaging as a tool to improve cancer screening programs (M-TICS Study): A randomized controlled trial protocol
Source: PLoS One. 2021 Jan 22;16(1):e0245806. doi: 10.1371/journal.pone.0245806 (PMC7822525; doi:10.1371/journal.pone.0245806)

**TITLE: Implementation of Text Messaging (SMS) as an Improvement Tool in Population-based Cancer Screening Programs**

Principal Investigator: MONTSERRAT GARCÍA MARTÍNEZ

ABSTRACT

The aim of this project is to assess the impact on health and economics of the implementation of text messaging (SMS) in cancer screening programs. Three interventions with SMS will be evaluated through community trials. In the colorectal cancer screening program the following interventions will be tested: a) Participation reminder: six weeks after sending the invitation letter of the colorectal cancer program and if there has not been a response, a reminder SMS will be sent in front of the usual method by letter; b) Reminder to return the fecal occult blood test: SMS reminder of test delivery versus no intervention. This reminder will be sent to the individuals who have gone to the pharmacy to pick up a fecal occult blood test and they have not returned it after 14 days. The impact on participation will be analyzed and, if applicable, the proportion of advanced neoplasm will be calculated by increase in participation. In the breast cancer screening program, the invitation by SMS versus the usual invitation by letter will be studied in women who had participated in the previous screening round. The impact on participation will be analyzed. A cost-effectiveness analysis of the three interventions will be carried out. The incremental cost ratio of the interventions between cost variation and effectiveness variation will be calculated.

BACKGROUND/ STATE OF THE ART

Purpose of the project

Cancer screening programs base their benefit on identifying a specific disease in an asymptomatic phase. For this purpose, they use some test to screen which individuals have a higher probability of having the disease. Currently, eligible population is invited to participate in those programs by mail. In recent decades, technologies of information and communication have been introduced into everyday life [1]. E-mail and mobile phone have been established as the main channels of communication of many people, displacing traditional postal mail or landline phone.

The field of health has been no exception to this reality, health systems are in a period of transition towards a Networked Society [2-4].

Text-messaging (SMS) allows communication through messages sent via mobile phone to a large number of individuals, at a relatively low cost and is delivered almost instantly. It is a less intrusive method than a phone call and can be read whenever is convenient for the receiver/recipient [5]. SMS facilitates communication with people living in areas where access to postal mail is limited. In addition, with text-messaging it is not necessary to have an up-to-date address in health records, as in the case with postal mail. SMS would allow improving accessibility to screening in population group with a high geographic mobility, which may be excluded from the screening programs due to frequent changes of address. Finally, it must be considered that among individuals with a high geographic mobility, there are vulnerable population groups [6].

Colorectal Cancer Screening Program – Catalan Institute of Oncology

The Catalan Institute of Oncology (ICO) manages a biennial population-based screening program for colorectal cancer (CRC) using fecal immunochemical occult blood test (FIT), which is provided free of charge. The target population, which is around 500,000 individuals, includes all men and women aged 50 to 69 years who live in the Metropolitan Area of Barcelona.  Demographic data on this population is gathered from the Primary Healthcare Information System. The screening area is divided into 96 Primary Healthcare Areas and screening invitations are sent to eligible population assigned to each one of the Primary Healthcare Areas. An invitation letter accompanied with an informative leaflet about the screening program and a list of the nearest pharmacies participating in the program is sent to the target population. Pharmacies provide, collect and sent FIT kits to a central laboratory.

Participation in the CRC screening program of ICO is lower than the standard indicated in the European Guidelines for Quality Assurance in CRC Screening that considered as acceptable participation of the 45% and as desirable 65%. [7] Therefore, it is necessary to implement measures that favor higher participation of the population. Besides overall low participation, it has been observed that in some population groups participation is even lower (people <60 years old, men and those with a lower socioeconomic level). [8-9]

Few studies have evaluated the impact of sending SMS reminders to participants in CRC screening programs that use FIT kit.[10] A study in the UK did not observe an overall increase in participation, but sending an SMS reminder did increase the number of first-time participants.[11] Studies in Israel observed increased participation among the population of lower socioeconomic status.[12,13] Adherence to (continued participation in) the CRC screening program of the ICO is very high (≥85%); therefore, it would be important to increase participation in people who are invited for the first time and/or those in younger age groups because this could improve the effectiveness of the program (population-wide impact on health). Further, if more people in socially depressed populations participated, health inequity would be reduced by improving accessibility to the CRC screening program.

Our group has conducted a pilot study of sending SMS reminders to test the viability of the intervention in two primary healthcare areas (unpublished data). In our program, people interested in participating in CRC screening have to go to a pharmacy to pick up and return their FIT kit. The pilot study has shown that it is feasible to send SMS reminders for the screening invitation. The preliminary results show that SMS is equal to or better than reminder letters in garnering participation. Phone calls were made to 150 people who had been sent the SMS to assess their understanding of the text message. Eighty percent of the users were aware of having received the message, and of these, 91% correctly identified the purpose of the message. Given the difficulty of implementing interventions that significantly increase participation in CRC screening and given the goal of keeping costs low (each and every health action must not only be evaluated in terms of health impact but in economic terms), it would be useful to identify population subgroups with greater motivation to participate in the program. Once these are identified, specific interventions should be designed and implemented for them. We have identified that in a period of two years (2017-2018), 13,000 FIT kits were collected in the collaborating pharmacies of the program that were not delivered for analysis. It can be assumed that these people are motivated for change (preparation phase), and a small SMS reminder to deliver the test could affect participation. [14.15]

Breast Cancer Screening Program – Catalan Institute of Oncology

The breast cancer (BC) program of the ICO coordinates the program of the southern metropolitan area of Barcelona and has a target population of approximately 160,000 women. Although it is widely accepted by the target population, there are several areas whose participation is lower than average; these are areas of new urban development and dispersed rural centers to which traditional mail is not delivered but is collected through the PO box system. Generally, there are a no negligible percentage of no-shows and subsequent rescheduling phone calls. Both the low participation of some specific areas and the general increase in telephone contacts are partly motivated by the difficulty and even loss of the habit of picking up traditional mail. The main objective of implementing new communication channels in the breast screening program would be to achieve, above all, better use and management of resources. Our group evaluated the usefulness of sending a reminder SMS 72 hours before a patient’s mammography appointment through a quasi-experimental study that compared a new invitation strategy (letter + SMS reminder) with the standard strategy (invitation letter). The text message was two-way, and a response was requested in case of cancellation or the need to reschedule the mammogram. [16] The SMS reminder led to greater participation in screening (OR: 1.56; 95% CI: 1.43-1.70). The increase in participation was higher among previous nonparticipants who lived in areas where access to postal mail is limited (OR: 2.85; 95% CI: 2.31-3.53) than in those who lived in areas of easy postal access (OR: 1.66; 95% CI: 1.36-2.02). On the other hand, the SMS was not useful as an appointment change tool because the women who selected the option to change the appointment could not choose the day and time of the mammogram; the new appointment was generated automatically. The program only allows you to change the appointment by choosing a new date through a website or by phone. Both options are widely used. In addition, women interested in having a mammogram and want to change the appointment usually contact the program as soon as they receive the invitation letter. There are more studies that have evaluated the incorporation of SMS as a means of contacting participants in BC programs. In general, an increase in participation has been observed that has allowed better scheduling management of the radiological units. [17-20]

Economic evaluation

Health technology economic evaluation examines the impact of a new health intervention on the state of health of their users by comparing it, in terms of cost, to a previous intervention. The incremental cost-effectiveness value between cost and effectiveness variation allows determining the extra cost per additional unit of output in order to assist decision-makers with establishing health priorities. The recommendation of the relevance of the analyzed technology is based on the efficiency threshold, which is over 30,000 Euros per year, adjusted for quality of life (QALY). [21] The study of costs differentiates between health costs (direct and future), non-health costs (supported by public authorities, patients and partners) and the cost of transfers (caused by monetary transactions between individuals and from the public sector, with no consumption of resources). [22] Typically, due to the difficulty of including a social perspective, cancer screening programs have been evaluated from the perspective of the funders [23, 24] and neither future costs nor transfer costs have been included.

REFERENCES

1 Atun RA, Stitampalam S. A review of the characteristics and benefits of SMS in delivering healthcare. In: Atun RA, et al editor(s). The role of cell phones in increasing accessibility and efficiency in healthcare. Vodafone Group PLC, 2006.

2 Free C, et al. The effectiveness of M-health tecnologies for improving Health and Health services: a systematic review protocol. BMC Res Notes. 2010; 3:250.

3 Krishna S, et al. Healthcare via Cell Phones: A Systematic Review. Telemed J E Health. 2009; 15:3.

4 Riley TW, et al. Health behavior models in the age of Mobile interventions: are our theories up to the task? Transl Behav Med. 2011: 1:53-71.

5 Mougalian SS,et al. Text Messaging in Oncology: A Review of the Lanscape. JCO Clin Cancer Inform. 2018; 2:1-9.

6 Vodopivec-Jamsek V, et al. Mobile phone messaging for preventive health care. Cochrane Database Syst Rev. 2012;12:CD007457.

7 Segnan N, Patnick J, von Karsa L (eds) European guidelines for quality assurance in colorectal cancer screening and diagnosis - First edition. European Commission, Publications Office of the European Union, Luxembourg, 2010.

8 Garcia M et al. Factors associated with initial participation in a population-based screening for colorectal cancer in Catalonia, Spain: a mixed-methods study. Prev Med. 2011, 52: 265-7.

9 Klabunde C, et al. Participation rates for organized colorectal cancer screening programs: an international comparison. J Med Screen. 2015;22:119-26.

10 Uy C, et al. Text Messaging Interventions on Cancer Screening Rates: A Systematic Review. JMIR. 2017; 19:e296.

11 Hirts Y, et al. Text-message Reminders in colorectal Cancer Screening (TRICCS): a randomised controlled trial. British Journal of Cancer. 2017; 116:1408-14.

12 Hagoel L, et al. Harnessing the question-behavior effect to enhance colorectal cancer screening in an mHealth experiment. Am J Public Health. 2016;106:1998-2004.

13 Hagoel L, et al. Better Ask Than Tell: Responses to mHealth Interrogative Reminders and Associations With Colorectal Cancer Screening Subsequent Uptake in a Prospective Cohort Intervetion. JMIR Mhealth Uhealth. 2019; 7: e9351.

14 Prochaska JO, Diclemente CC. Stages of change in the modification of problema behaviors. Prog Behav Modif. 1992;28:183-218.

15 Costanza ME, et al. Applying a stage model of behavior change to CRC screening. Prev Med. 2005;41:707-19.

16 Vidal C, et al. Use of text-message reminders to improve participation in a population-base breast cancer sreening program. J Med Syst. 2014;38:118.

17 Guy R, et al. How effective are short message service reminder at increasing clinic attendance? A meta-analysis and systematic review. Health Serv Res. 2012;47;614-32.

18 Gurol-Urganci I, et al. Mobile phone messaging reminders for attendance at healthcare appointments. Cochrane Database Syst Rev. 2013;12: CD007458

19 Lakkis AN, et al. The effect of two types of sms-texts on the uptake of screening mammogram: A randomized controlled trial. Prev Med. 2011; 53: 325-7.

20 Kerrison SR, et al. Text-message reminders increase uptake of routine breast srceening appointments: a randomised controlled t

21 Sacristán JA, et al. ¿Qué es una tecnología eficiente en España? Gac Sanit.2002;16:334-43.

22 Puig-Junoy J, et al. Los costes en la evaluación económica de tecnologías sanitarias. Aten Primaria.2001;3:186-9.

23 Carles M, et al. Cost-effectiveness of early detection of breast cancer in Catalonia (Spain). BMC Cancer.2011;11:192.

24 Arrospide A, et al. Cost-effectiveness and budget impact analysis of a colorectal cancer screening programme in a high adenoma prevalence scenario using MISCAN-Colon microsimulation model. BMC Cancer. 2018:18:464.

HYPOTHESIS

The implementation of SMS in population-based cancer screening programs will improve participation, especially in CRC screening, will allow better management of resources, and will reduce costs in both programs.

Specific hypotheses:

Sending SMS reminder will have a greater impact on participation than the standard reminder (letter) in the CRC screening program.

The effect of SMS reminder on CRC screening will be greater among individuals who have been invited for the first time and who have not previously participated.

Participation after receiving SMS invitation in BC screening will be equal to or better than the invitation by letter.

The option that includes the sending of SMS will be cost-effective in comparison to the current method used.

OBJECTIVES

Main Objective

To evaluate the effectiveness of SMS in population-based cancer screening programs in the Metropolitan Area of Barcelona.

Specific objectives

To analyze the effect of SMS reminders in a CRC screening program.

To analyze the effect of SMS reminder on participation in a CRC screening program by previous screening behavior (non-participants, new participants, previous participants) and residing in areas of limited postal access.

To assess the impact on participation and cost reduction of SMS FIT return reminder in a CRC screening program.

To analyze the impact on successive participation of receiving an invitation to the BC screening program by SMS.

To evaluate the usefulness of SMS as an appointment re-scheduling tool for screening mammograms.

To analyze the cost-effectiveness of the three SMS interventions, two in the CRC screening program and one in the breast screening program in relation to the current intervention.

INTERVENTIONS

Intervention 1: Invitation reminder for CRC screening.

Design and study population

Cluster randomized controlled trial. Men and women between 50 and 69 years of age from eight primary healthcare areas will be invited to CRC screening. Invitations to the program will be sent weekly. The invitation weeks (clusters) will be randomized so that there will be weeks in which the individuals whose mobile phones we have registered will receive the reminder by letter, and other weeks they will receive the reminder by SMS. The rest of the individuals, for whom we do not have mobile phone information (15%), will receive the reminder by letter as usual. These individuals will not enter the analysis that will compare the letter and the SMS as a reminder. The reminder will be sent to individuals who have not completed the FIT kit 6 weeks into their screening episode, corresponding to approximately 70% of the cases.

Variables

The dependent variable will be participation in screening (yes/no). This variable will be collected prospectively after participants are invited to the screening program through a database connected to the screening program registry. Participation will be analyzed from week 18 after mailing out the invitation letter (week 0). The independent variables that will be collected are the type of reminder (SMS/letter), previous screening status (first-time invited, nonparticipants, participants in the previous round), and socio-demographic variables such as age, sex, and the socioeconomic deprivation index developed by the Agency for Health Quality and Assessment of Catalonia (AQuAS). It is an aggregate index by primary healthcare area that includes the following variables: percentage of manual laborers, percentage of people with insufficient education, and premature mortality in individuals <75 years, avoidable hospitalization rate and population served in primary care). Values range from 0-100, with higher values implying lower socioeconomic levels.

Finally, a variable will be calculated on the accessibility of postal mail (easy access areas/hard to reach areas). This variable will be calculated considering the population growth in the last 10 years and the proportion of municipalities with less than 15,000 inhabitants. Vidal et al, 2014

Sample size

The sample size (n=10,415) has been calculated to identify differences according to previous screening of 3%. The distribution of our study population by screening is: 14% new invitees, 53% non-participants and 33% participants in the previous round. An alpha risk of 5% and a statistical power of 80% have been considered. In addition, 10% of wrong registered phones have been estimated.

Data analysis

A multivariate analysis will be performed using a logistic regression model with random intercept to compare the experimental intervention (SMS) versus the standard intervention (letter). The "invitation week" will be included in the model to account for cluster variability. Odds ratios and 95% confidence intervals will be calculated.

Intervention 2: SMS for FIT delivery in CRC screening

Study design and population

Cluster randomized controlled trial on all individuals with a mobile phone number registered who have picked up a FIT kit and has not been returned at the pharmacy within 14 days from pick-up. This 14-day time interval has been considered since 90% of the people deliver the screening test within this period and to avoid, moreover, that they have misplaced it. The duration of the study will be 12 months.

Sample size

The basic health areas invited in one year (n=47) will be randomly assigned to the two groups: 1) Experimental group in which an SMS reminder for delivery of FIT will be designed and implemented; 2) This study will have a statistical power of 92% to detect differences in participation of 3% between the experimental group and the control group.

Variables

The dependent variable will be the participation in CRC screening. Participation will be analyzed at 8 week after sending the invitation letter (week 0). The independent variables will be: Intervention (SMS/no intervention), previous screening behavior (new participants, non-participants, participants in the previous round) and socio-demographic variables: age, sex and the socio-economic deprivation index (AQUAS).

Data analysis

A multivariate analysis will be performed using a random intercept logistic regression model to compare the control group (SMS reminder of FIT delivery) and the control group (no intervention). The core health area will be included as a random effects intercept. Odds ratios and 95% confidence intervals will be calculated.

Intervention 3: Screening invitation for successive participants in BC screening

Design and Study population

Randomized controlled trial. All women aged 52-69 who have participated in the BC screening program in the immediately preceding round and have a mobile phone number registered in the HealthCare Database.

Sample size

About 80,000 women are invited each year, half of whom participated in the previous round. As the invitation in the program is not made by basic health area but by date of birth, the assignment to the intervention groups will be made by a simple random allocation. Women whose mobile number is not available will receive the invitation letter but will not be included in the study. An interim analysis will be performed two months after the start of the trial, and the trial will be stopped if timely participation in the group invited by SMS compared to the guest by letter is reduced by 3% or more.

Variables

The dependent variable will be participation or not in the screening 8 weeks after the initial date of invitation to the program. Invitation (SMS/letter) will be independent variable and other variables will be collected: previous screening behavior, accessibility to postal mail and socio-demographic variables (age and AQUAS-social index).

Analysis

Logistic regression models will determine if the SMS invitation has an equal or better participation than the letter invitation.

Process evaluation

All three interventions will include a process evaluation. We will use a mixed-methods study design, including a survey (n=100 for each intervention) to test reading and understanding of text messages among participants, and qualitative interviews to explore participants’ experiences of the interventions (with an estimated sample of n=36 for interventions 1 and 2, and n=24 for intervention 3). Findings from the process evaluation will inform interpretation of outcomes and any variation of effects in subgroups as well as strategies to refine and optimize the tested interventions.

Cost-effectiveness analysis

The cost-effectiveness analysis will be done through a Decision Tree for each of three interventions that will take into account the use of SMS on costs and results.

Healthcare financer perspective will be analyzed. Following healthcare costs will be included, which will be evaluated in the population-based screening programs: a) Labor costs, consumables and depreciation of property, facilities and equipment, as well as "overheads". The SMS alternative will specifically include the personnel and infrastructure fixed costs; b) Diagnosis costs, specifically the costs of evaluating cases with a positive screening test. Future costs will not be included.

The effectiveness will be measured through the number of people who have participated in the screening and, if applicable, the proportion of advanced malignancies detected by increased participation will be calculated.

The result will be specified as an incremental ratio between costs and effectiveness (ICER) which was defined as cost per participants. The temporary period of analysis will be 1 year. A deterministic sensibility analysis will be applied to test the robustness of the results.

LIMITATIONS

Among the limitations of the study, we highlight that in order to incorporate SMS as a new communication tool; the proportion of users with a correct number must be high. We are able to access the mobile phone number of more than 85% of the eligible population and this proportion is increasing year after year.

In addition, in two previous studies that we carried out in both BC and CRC cancer screening, the proportion of wrong numbers was 9-10%.

The size of the SMS might be a limitation, as it has to be limited to 160 characters. However, we can add a link to more detailed information on the screening process. Example: ICO reminds you of the importance of preventing RCC. If you want to take the test, go to: <https://zmz.es/dKmBlg>

Ethical aspects

The Catalan Institute of Oncology the entity responsible for the processing of personal data of men and women between 50 and 69 years of age in the southern metropolitan area of Barcelona invited to participate in population programs for the early detection of cancer, is in the process of adjusting and updating its protocols, arrangements, conventions, terms, ..., to respond adequately to new regulations on confidentiality and protection of personal data (Regulation 2016/679 of the European Parliament and Council of 27 April 2016 on the protection of individuals (RGPD) and Law Organic Law 3/2018 of 5 December on the Protection of Personal Data and Guarantee of Digital Rights (LOPD-GDD).

The database, with prospective information, will have a coded identification with restricted access. The statistical analysis will be carried out on individual information but will not allow the identification of the subjects. The crossing of the different databases will be subject to statistical confidentiality.

Developing steps and assignment of tasks for the complete research team, and the planned assignments for the technical staff requested. Also indicate the place/centre where the project is to be performed

(1) Adaptation of the software applications of screening programs: It is planned to contract external services (computer companies) to implement the changes required in the software applications of the screening programs to enable SMS invitation or reminder. Setting: external companies.

2) Planning CCR screening interventions and subsequent presentation to collaborating pharmacies. The research team will be involved in the development of text messages content in both the invitation reminder of participation and the FIT return reminder. The distribution will be planned and the procedures to be followed in the case of incidents with collaborating pharmacies will be determined. It will be hold informative meetings with pharmacies in each of the primary healthcare areas selected for the study. Setting: ICO, Official College of Pharmacists of Barcelona.

3) Planning BC screening interventions. The research team will be involved in the development of text messages content. Setting: ICO.

4) Field research: Developing interventions. The administration team of screening programs will have an important role in monitoring incidents as they are the first contact with both users and pharmacies. In addition, a FP Technician will be hired for the coordination and monitoring of the interventions together with Montse Garcia. Coordination and monitoring setting: ICO.

5) Process evaluation: Data on the process of the interventions will be collected to capture the perspectives and experiences of the participants through a mixed-method strategy. The data collection will take place at several time points since the SMS were sent for each of the three interventions evaluated. Albert Farré, Montse Garcia. Setting: ICO, University from Dundee.

6) Development and execution of the analysis plan: Once field research is completed the information will be analyzed. The aim is to assess the association of the variables of interest and its effect on participation. Núria Milà, Noémie Travier and Montse Garcia will participate in this stage of the project. Setting: ICO.

(7) Cost-effectiveness analysis: direct health costs of screening programs will be collected. A literature search will be conducted to obtain non-health costs. Maria José Pérez, Montse Garcia. Setting: ICO.

8) Interpretation, discussion and dissemination of results: The research team will actively participate in the discussion and interpretation of the results and will develop the plan for Dissemination of results (main targets: managers, researchers, opinion leaders and general population) Plan will include press presentations, conference presentations, publications scientific, reports to the administration and mass media... Setting: ICO.

Chronogram


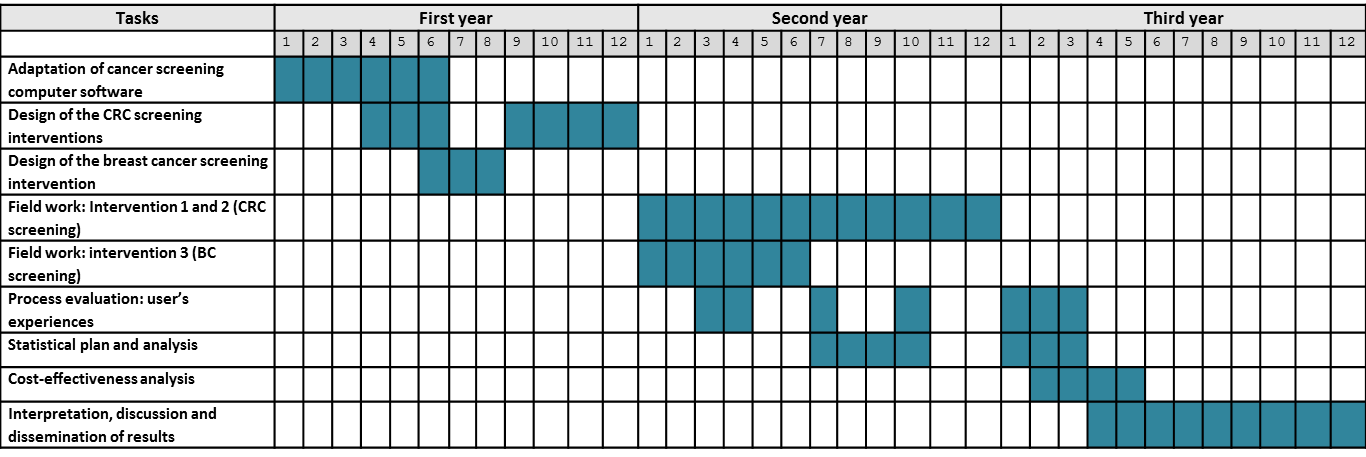

Supplement: S1 File — (DOCX) [file pone.0245806.s003.docx]
