# Supplementary material for: Text messaging as a tool to improve cancer screening programs (M-TICS Study): A randomized controlled trial protocol
Source: PLoS One. 2021 Jan 22;16(1):e0245806. doi: 10.1371/journal.pone.0245806 (PMC7822525; doi:10.1371/journal.pone.0245806)

Expediente Nº PI/00226

TITULO: **Implementación de la mensajería de texto (SMS) como herramienta de mejora en los programas poblacionales de cribado de cáncer**

INVESTIGADOR/A PRINCIPAL: MONTSERRAT GARCÍA MARTÍNEZ

CO-INVESTIGADOR/A PRINCIPAL:

TIPO DE PROYECTO: (●) INDIVIDUAL ( ) COORDINADO ( ) MULTICÉNTRICO

NOMBRE DEL IP COORDINADOR:
(Cumplimentar sólo en caso de proyectos coordinados)

DURACIÓN: ☒ **3 AÑOS**

RESUMEN (Objetivos y Metodología del Proyecto) **(Ajústese al espacio disponible)**

La finalidad de este proyecto es evaluar el impacto en salud y económico de la implantación de mensajería de texto (SMS) en los programas de cribado de cáncer. Se evaluarán tres intervenciones con SMS mediante ensayos comunitarios. En el **programa de cribado cáncer colorrectal** se testarán las siguientes intervenciones: a) Recordatorio para la participación: a las seis semanas de haber enviado la carta de invitación del programa de cáncer colorrectal y si no ha habido respuesta, se enviará un SMS recordatorio frente al método habitual por carta; b) Recordatorio de la entrega del test de sangre oculta en heces una vez recogido: SMS recordatorio de entrega de prueba versus no intervención. Este tipo de recordatorio se enviará a las personas que hayan acudido a la farmacia a recoger un test de sangre oculta en heces y que a los 14 días no lo hayan entregado. Se analizará el impacto en participación y, si procede, se calculará la proporción de neoplasias avanzadas por incremento en participación. En el **programa de cribado de cáncer de mama** se estudiará el envío de invitación por SMS versus la invitación habitual por carta en las participantes sucesivas regulares (participación previa en la ronda anterior) en el programa. Se analizará el impacto en participación. Se realizará un análisis de coste-efectividad de las tres intervenciones. Se calculará la ratio de coste incremental de las intervenciones entre variación de costes y variación de efectividad.

TITLE: Implementation of text messaging (SMS) as an improvement tool in population-based cancer screening programs

ABSTRACT (Objectives and Methodology of the Project) **(Please only use the space provided below)**

The aim of this project is to assess the impact on health and economics of the implementation of text messaging (SMS) in cancer screening programs. Three interventions with SMS will be evaluated through community trials. In the **colorectal cancer screening program** the following interventions will be tested: a) Participation reminder: six weeks after sending the invitation letter of the colorectal cancer program and if there has not been a response, a reminder SMS will be sent in front of the usual method by letter; b) Reminder to return the fecal occult blood test: SMS reminder of test delivery versus no intervention. This reminder will be sent to the individuals who have gone to the pharmacy to pick up a fecal occult blood test and they have not returned it after 14 days. The impact on participation will be analyzed and, if applicable, the proportion of advanced neoplasms will be calculated by increase in participation. In the **breast cancer screening program**, the invitation by SMS versus the usual invitation by letter will be studied in women who had participated in the previous screening round. The impact on participation will be analyzed. A cost-effectiveness analysis of the three interventions will be carried out. The incremental cost ratio of the interventions between cost variation and effectiveness variation will be calculated.

Expediente Nº PI/00226

INVESTIGADOR/A PRINCIPAL: MONTSERRAT GARCÍA MARTÍNEZ

MEMORIA DE SOLICITUD PROYECTO DE INVESTIGACIÓN EN SALUD
SECCIÓN ANTECEDENTES Y ESTADO ACTUAL DEL TEMA

Finalidad del proyecto, antecedentes y estado actual de los conocimientos científico-técnicos, grupos nacionales o internacionales que trabajan en la línea específica del proyecto o en líneas afines.
Citar las referencias en el apartado siguiente: Bibliografía más relevante. **Máximo 3 páginas (15.700 caracteres)**

Finalidad del proyecto

Los programas de detección precoz fundamentan su beneficio en la identificación de una enfermedad concreta en fase asintomática, para ello se sirven de recursos que proveen una determinada prueba diagnóstica a grupos de población en riesgo de padecerla. Actualmente, la forma de invitar a la población elegible a participar en los programas de cribado es por correo postal. En las últimas décadas las tecnologías de la información y la comunicación se han introducido en la vida cotidiana tanto en el ámbito económico como en el social. [1] El e-mail o la telefonía móvil se han establecido como las principales vías de comunicación de muchas personas, desplazando al tradicional correo postal o a la telefonía fija. El ámbito de la salud no es ajeno a esta realidad, los sistemas de salud están en un período de transición hacia la Sociedad en Red. [2-4]
La mensajería de texto, short messaging service (SMS), permite comunicarse mediante mensajes enviados a través de la telefonía móvil a un gran número de individuos, a un coste relativamente bajo y son entregados casi al instante. Es un método menos intrusivo que una llamada telefónica y se pueden leer siempre que sea conveniente para el receptor. [5] El SMS facilita la comunicación con personas que viven en zonas de difícil acceso al correo postal; además, con este sistema no es necesario tener una dirección estable y actualizada en los registros sanitarios como ocurre en el caso del correo postal, con lo que permitiría mejorar la accesibilidad a los cribados en población con alta movilidad geográfica que por sus cambios frecuentes de domicilio pueden quedarse al margen de estos programas. Finalmente, se debe tener en cuenta que entre la población con alta movilidad geográfica existen grupos de población vulnerable. [6]

Programa de Detección Precoz de Cáncer Colorrectal - Instituto Catalán de Oncología

El programa de cribado de cáncer colorrectal (CCR) del Instituto Catalán de Oncología (ICO) se implementó en el año 2000 en Hospitalet de Llobregat, siendo el primer programa poblacional de detección precoz de CCR en España. En el año 2004, se amplió el programa a Vilafranca del Penedés y posteriormente se incluyó a toda la población de la comarca del Alt Penedés (2007). A partir del año 2015, el ICO asumió la extensión del programa al área Metropolitana de Barcelona alcanzando una población total de 474.000 personas, hombres y mujeres entre 50 y 69 años, lo que supone la implicación de 94 áreas básicas de salud y la colaboración con 608 farmacias y 11 unidades de endoscopia.
La participación en el programa poblacional de cribado de CCR es inferior al estándar indicado por la Guía Europea de Calidad de Programas de Cribado de CCR que consideró como aceptable una participación del 45% y como deseable del 65%. [7] Por tanto, es necesario implementar medidas que favorezcan una mayor participación de la población. Además de la baja participación global, se ha observado que en algunos grupos de población la participación aún es menor (personas <60 años, hombres y aquellos con un nivel socioeconómico más bajo). [8-9]

Pocos estudios han evaluado el impacto en participación del envío de SMS recordatorios en programas de cribado de CCR mediante test de sangre oculta en heces (TSOH). [10] Un estudio realizado en el Reino Unido no observó un aumento global de la participación, pero sí que el envío de un SMS recordatorio aumentaba entre los individuos que habían sido invitados por primera vez. [11] En estudios realizados en Israel se

observó un aumento de participación entre la población de menor nivel socioeconómico. [12,13]
La adherencia (participación continuada) del programa de cribado de CCR del ICO es muy alta (≥85%), por tanto, sería importante aumentar la participación en personas que son invitadas por primera vez y/o en los grupos de edad más jóvenes ya que podría mejorar la efectividad del programa (impacto poblacional en salud). Por otro lado, si se demostrase un aumento en la participación en poblaciones socialmente más deprimidas se reduciría la inequidad en salud al mejorar la accesibilidad al programa de cribado de CCR.
Nuestro grupo ha realizado un estudio piloto de envío de recordatorios mediante SMS para testar la viabilidad de la intervención en dos áreas básicas de salud (datos no publicados). En nuestro programa, las personas interesadas en participar en el cribado de CCR han de acudir a una farmacia tanto para recoger como para entregar el TSOH. El estudio piloto ha demostrado que es factible la implementación del SMS como recordatorio de la invitación al cribado. Los resultados preliminares muestran que la participación suscitada por el SMS es igual o mejor que el recordatorio mediante carta. Se realizaron llamadas telefónicas a 150 personas que se les había enviado el SMS para valorar la comprensión del mensaje de texto. El 80% de los usuarios eran conocedores de haber recibido el mensaje y de éstos el 91% identificaron correctamente cuál era la finalidad del mensaje.

Dada la dificultad de implementar intervenciones que aumenten de forma significativa la participación en cribado de CCR y en un contexto general de contención del gasto, en las que todas y cada una de las acciones sanitarias no sólo han de ser evaluadas en términos de impacto en salud, sino en términos económicos, sería útil poder identificar subgrupos de población con una mayor motivación a participar en el programa. Una vez identificados, se debería diseñar e implementar intervenciones específicas para ellos. Hemos identificado que en un período de dos años (2017-2018) se recogieron 13.000 TSOH en las farmacias colaboradoras del programa que no fueron entregados para su análisis. Podríamos considerar que estas personas están motivadas para el cambio (*fase de preparación*) y un pequeño recordatorio SMS de entrega de la prueba podría incidir en la participación. [14,15]

Programa de Detección Precoz de Cáncer de Mama - ICO
El programa de cáncer de mama del ICO coordina el programa del área metropolitana sur de Barcelona, tiene una población diana aproximada de 160.000 mujeres. Aunque es un programa consolidado de gran aceptación por parte de la población diana, se han detectado zonas donde la participación es más baja que la media del territorio, se trata de áreas de nuevo desarrollo urbanístico y de núcleos rurales dispersos a los que el correo tradicional no llega y, la recogida se realiza mediante el sistema de apartados de correo. En general en el programa existe un porcentaje no desdeñable de incomparecencias y de llamadas posteriores solicitando una nueva cita. Tanto la baja participación de algunas zonas concretas, como el incremento general de contactos telefónicos se considera que puede estar en parte motivado por la dificultad e incluso por la pérdida de costumbre de recoger el correo tradicional. El objetivo principal de implementar nuevas vías de comunicación en el programa de mama sería lograr, sobre todo, una mejor utilización y gestión de los recursos.

Nuestro grupo evaluó la utilidad del envío de un SMS recordatorio 72 horas antes de la cita de la mamografía mediante un estudio cuasi-experimental que comparaba una nueva estrategia de invitación (carta + recordatorio SMS) con la estrategia estándar (carta de invitación). El mensaje de texto era bidireccional, se solicitaba respuesta en caso de no asistencia o necesidad de cambiar el día y hora de la mamografía. [16]

El recordatorio mediante SMS suscitó un aumento de la participación en el cribado (OR:1,56; IC95%:1,43-1,70). El incremento en participación fue mayor entre las no participantes previas y que vivían en áreas donde el acceso al correo postal es limitado (OR:2,85; IC95%:2,31-3,53) comparado con las que vivían en zonas de fácil acceso postal (OR:1,66; IC95%:1,36-2,02). En cambio, el SMS no fue útil como

herramienta de cambio de cita porque las mujeres que seleccionaban la opción cambiar de cita no podían elegir el día y hora de la mamografía, la nueva cita se generaba automáticamente. El programa solo permite cambiar la cita eligiendo una nueva fecha a través de una página web o bien telefónicamente. Ambas opciones son ampliamente utilizadas. Además, las mujeres interesadas en realizarse la mamografía y que desean cambiar la cita suelen ponerse en contacto con el programa en cuanto reciben la carta de invitación.

Existen más estudios que han evaluado la incorporación de SMS como vía de contacto entre las usuarias de los programas de cáncer de mama. En general, se ha observado un incremento de la participación que ha permitido una mejor gestión de la programación de las agendas de las unidades radiológicas. [17-20]

Evaluación económica

La evaluación económica de tecnologías sanitarias estudia el impacto de una nueva intervención sanitaria sobre el estado de salud de los usuarios comparándola, en términos de costes, con una intervención previa. El valor de la ratio incremental coste-efectividad entre variación de costes y variación de efectividad permite conocer el coste adicional por cada unidad adicional de resultado y ayuda a los decisores a establecer prioridades sanitarias. La recomendación de la aplicabilidad o no de la tecnología analizada se determina en función del umbral de eficiencia que se sitúa sobre los 30.000 euros por año ganado ajustado por calidad de vida (AVAC). [21] El estudio de costes distingue entre costes sanitarios (directos y futuros), costes no sanitarios (soportados por presupuestos públicos, pacientes y acompañantes) y coste de transferencias (producidos por los flujos monetarios entre individuos o bien desde el sector público, sin consumo de recursos). [22] Habitualmente, debido a la dificultad de considerar la perspectiva social, los programas de cribado de cáncer se han evaluado desde la perspectiva del financiador [23,24] y no se han incluído ni los costes futuros ni los costes de transferencias.

Expediente Nº PI/00226

INVESTIGADOR/A PRINCIPAL: MONTSERRAT GARCÍA MARTÍNEZ

MEMORIA DE SOLICITUD PROYECTO DE INVESTIGACIÓN EN SALUD
SECCIÓN ANTECEDENTES Y ESTADO ACTUAL DEL TEMA

Citar las referencias incluidas en el apartado anterior: Antecedentes y Estado actual. (Máximo 1 página)

1 Atun RA, Stitampalam S. A review of the characteristics and benefits of SMS in delivering healthcare. In: Atun RA, et al editor(s). The role of cell phones in increasing accessibility and efficiency in healthcare. Vodafone Group PLC, 2006.

2 Free C, et al. The effectiveness of M-health tecnologies for improving Health and Health services: a systematic review protocol. BMC Res Notes. 2010; 3:250.

3 Krishna S, et al. Healthcare via Cell Phones: A Systematic Review. Telemed J E Health. 2009; 15:3.

4 Riley TW, et al. Health behavior models in the age of Mobile interventions: are our theories up to the task? Transl Behav Med. 2011: 1:53-71.

5 Mougalian SS,et al. Text Messaging in Oncology: A Review of the Lanscape. JCO Clin Cancer Inform. 2018; 2:1-9.

6 Vodopivec-Jamsek V, et al. Mobile phone messaging for preventive health care. Cochrane Database Syst Rev. 2012;12:CD007457.

7 Segnan N, Patnick J, von Karsa L (eds) European guidelines for quality assurance in colorectal cancer screening and diagnosis - First edition. European Commission, Publications Office of the European Union, Luxembourg, 2010.

8 Garcia M et al. Factors associated with initial participation in a population-based screening for colorectal cancer in Catalonia, Spain: a mixed-methods study. Prev Med. 2011, 52: 265-7.

9 Klabunde C, et al. Participation rates for organized colorectal cancer screening programs: an international comparison. J Med Screen. 2015;22:119-26.

10 Uy C, et al. Text Messaging Interventions on Cancer Screening Rates: A Systematic Review. JMIR. 2017; 19:e296.

11 Hirts Y, et al. Text-message Reminders in colorectal Cancer Screening (TRICCS): a randomised controlled trial. British Journal of Cancer. 2017; 116:1408-14.

12 Hagoel L, et al. Harnessing the question-behavior effect to enhance colorectal cancer screening in an mHealth experiment. Am J Public Health. 2016;106:1998-2004.

13 Hagoel L, et al. Better Ask Than Tell: Responses to mHealth Interrogative Reminders and Associations With Colorectal Cancer Screening Subsequent Uptake in a Prospective Cohort Intervetion. JMIR Mhealth Uhealth. 2019; 7: e9351.

14 Prochaska JO, Diclemente CC. Stages of change in the modification of problema behaviors. Prog Behav Modif. 1992;28:183-218.

15 Costanza ME, et al. Applying a stage model of behavior change to CRC screening. Prev Med. 2005;41:707-19.

16 Vidal C, et al. Use of text-message reminders to improve participation in a population-base breast cancer sreening program. J Med Syst. 2014;38:118.

17 Guy R, et al. How effective are short message service reminder at increasing clinic attendance? A meta-analysis and systematic review. Health Serv Res. 2012;47;614-32.

18 Gurol-Urganci I, et al. Mobile phone messaging reminders for attendance at healthcare appointments. Cochrane Database Syst Rev. 2013;12: CD007458

19 Lakkis AN, et al. The effect of two types of sms-texts on the uptake of screening mammogram: A randomized controlled trial. Prev Med. 2011; 53: 325-7.

20 Kerrison SR, et al. Text-message reminders increase uptake of routine breast srceening appointments: a randomised controlled t

21 Sacristán JA, et al. ¿Qué es una tecnología eficiente en España? Gac Sanit.2002;16:334-43.

22 Puig-Junoy J, et al. Los costes en la evaluación económica de tecnologías sanitarias. Aten Primaria.2001;3:186-9.

23 Carles M, et al. Cost-effectiveness of early detection of breast cancer in Catalonia (Spain). BMC Cancer.2011;11:192.

24 Arrospide A, et al. Cost-effectiveness and budget impact analysis of a colorectal cancer screening programme in a high adenoma prevalence scenario using MISCAN-Colon microsimulation model. BMC Cancer. 2018:18:464.

Expediente Nº PI/00226

INVESTIGADOR/A PRINCIPAL: MONTSERRAT GARCÍA MARTÍNEZ

MEMORIA DE SOLICITUD PROYECTO DE INVESTIGACIÓN EN SALUD
SECCIÓN HIPÓTESIS Y OBJETIVOS

(Ajústese al espacio disponible)

HIPÓTESIS

La implementación del SMS en los programas poblacionales de cribado de cáncer supondrá una mejora de la participación, especialmente en el cribado de CCR, permitirá una mejor gestión de los recursos y reducir costes en ambos programas.

Hipótesis específicas

El envío del SMS recordatorio tendrá un impacto en la participación mayor que el obtenido con el recordatorio estándar (carta) en el programa de cribado de CCR.

El efecto del SMS recordatorio del cribado de CCR será superior entre los individuos que han sido invitados por primera vez y no participantes anteriores.

La participación tras recibir la invitación por SMS en el cribado de cáncer de mama será igual o mejor que la invitación por carta.

La alternativa que incluye el envío de SMS será coste-efectiva en relación a la alternativa actual que utiliza el envío postal.

OBJETIVOS

Objetivo Principal

Evaluar la utilidad del SMS en los programas poblacionales de cribado de cáncer del Área Metropolitana de Barcelona

Objetivos Específicos

- Analizar el efecto en la participación en un programa de cribado de CCR del envío de recordatorios mediante SMS.
- Identificar si el efecto del SMS recordatorio del cribado de CCR es superior en función de la experiencia previa del cribado (no participantes anteriores, nuevas invitaciones, participantes sucesivos) y residir en áreas de limitado acceso postal.
- Evaluar el impacto en participación y reducción de costes del recordatorio de entrega de TSOH por SMS.
- Analizar el impacto en la participación sucesiva de la invitación al programa de cribado de cáncer de mama mediante SMS.
- Evaluar la utilidad de los SMS como herramienta de cambio de cita para las mamografías de cribado.
- Analizar la relación coste-efectividad de tres intervenciones con SMS, dos de ellas en el programa de cribado de CCR y una en el de mama, en relación a la actuación actual.

Expediente Nº
PI/00226

INVESTIGADOR/A PRINCIPAL: MONTSERRAT GARCÍA MARTÍNEZ

MEMORIA DE SOLICITUD PROYECTO DE INVESTIGACIÓN EN SALUD
SECCIÓN PROYECTOS COORDINADOS

En caso de Proyectos Coordinados, el COORDINADOR deberá indicar:
- Objetivos globales del proyecto coordinado, la necesidad de dicha coordinación y el valor añadido que se espera obtener de la misma.
- Objetivos específicos de cada subproyecto (deben estar recogidos además en la memoria de cada subproyecto)
- Interacción entre los distintos objetivos, actividades y subproyectos.
- Los mecanismos de coordinación previstos para la eficaz ejecución del proyecto. **Máximo 3 páginas (15.700 caracteres)**

Expediente Nº PI/00226

INVESTIGADOR/A PRINCIPAL: MONTSERRAT GARCÍA MARTÍNEZ

MEMORIA DE SOLICITUD PROYECTO DE INVESTIGACIÓN EN SALUD
SECCIÓN METODOLOGÍA
Diseño, sujetos de estudio, variables, recogida y análisis de datos y limitaciones del estudio.
Máximo 3 páginas (15.700 caracteres)

La figura 1 (**Anexos**) muestra el diagrama de flujo de las tres intervenciones del proyecto.

Intervención 1: SMS recordatorio en el cribado de CCR

Diseño y población de estudio: Ensayo aleatorizado por conglomerados. Hombres y mujeres entre 50 y 69 años de ocho áreas básicas de salud (n=10.415) invitados al cribado de CCR. Las invitaciones del programa se envían semanalmente. Se aleatorizarán las semanas de invitación (conglomerados) de manera que habrá semanas en los que los individuos cuyo teléfono móvil tenemos registrado recibirán el recordatorio por carta y otras semanas recibirán el recordatorio por SMS. El resto de individuos, de los que no somos conocedores de su teléfono móvil (15%), recibirán el recordatorio por carta, como es habitual. Esos individuos no entrarán en el análisis que comparará la carta y el SMS como recordatorio. El envío del recordatorio se realiza a las seis semanas en el caso de no haber recibido el TSOH para analizar y aproximadamente corresponde a un 70% de los casos.
Variables: La variable dependiente será la participación en el cribado (sí/no). Esta variable se recogerá de forma prospectiva tras la invitación al programa de cribado, mediante la conexión de la base de datos del registro del Programa de Cribado. Se analizará la participación a partir de la semana 18 después del envío de la carta de invitación (semana 0). Las variables independientes que se recogerán son el tipo de recordatorio (SMS/carta), el cribado previo (nuevas incorporaciones, no participantes, participantes en ronda anterior), variables sociodemográficas como la edad, el sexo y el índice de privación socioeconómica elaborado por la Agencia de Calidad y Evaluación Sanitarias de Cataluña (AQUAS). Es un índice agregado por área básica de salud que incluye las siguientes variables (% de trabajadores manuales, % de personas con estudios insuficientes, mortalidad prematura en individuos <75 años, tasa de hospitalización evitable y población atendida en atención primaria). El rango de valores oscila entre 0-100, los valores más elevados suponen niveles socioeconómicos más bajos.
Por último, se calculará una variable sobre la accesibilidad del correo postal (áreas de fácil acceso/áreas de difícil acceso) considerando el crecimiento poblacional en los últimos 10 años y la proporción de municipios con menos de 15.000 habitantes. [Vidal et al, 2014]
Muestra: El tamaño muestral (ocho áreas básicas de salud) se ha calculado para identificar diferencias según cribado previo del 3%. La distribución de nuestra población de estudio según cribado previo es: 14% de nuevas incorporaciones, 53% de no participantes y 33% de participantes en ronda anterior. Para el cálculo muestral se ha considerado un riesgo alfa del 5% y una potencia estadística del 80%. Además, se ha estimado un 10% de teléfonos erróneos.
Análisis de datos: Se realizará un análisis multivariante mediante un modelo de regresión logística de intercepción aleatoria para comparar la intervención experimental (SMS) versus la intervención estándar (carta). Se incluirá la "semana de invitación" en el modelo para tener en cuenta la variabilidad de los conglomerados. Se calcularán las Odds Ratios y los intervalos de confianza del 95%.

Intervención 2: SMS de entrega de TSOH en el cribado de CCR
Diseño y población de estudio: Ensayo aleatorizado por conglomerados
en los individuos con un número de teléfono móvil registrado en nuestra aplicación, que hayan recogido el TSOH en la farmacia y que a los 14 días todavía no lo hayan entregado. Se ha considerado ese intervalo de

tiempo de 14 días dado que el 90% de las personas entregan la prueba de cribado en ese período y para evitar, además, que la hayan extraviado. La duración del estudio será de 12 meses. Se asignarán las áreas básicas de salud invitadas en un año (n=47) de forma aleatoria a los dos grupos: 1. Grupo experimental en el que se diseñará e implementará un recordatorio SMS de entrega de TSOH; 2. Grupo control en el que no se realizará ninguna intervención. Este estudio tendrá una potencia estadística superior al 80% de detectar diferencias en participación del 3% entre el grupo experimental y el grupo control.
Variables: La variable dependiente será la participación en el cribado (sí/no). Las variables independientes que se recogerán son el tipo de intervención (SMS/no intervención), el cribado previo (nuevas incorporaciones, no participantes, participantes en ronda anterior) y variables sociodemográficas (edad e índice de privación socioeconómica AQUAS).
Análisis de datos: Se realizará un análisis multivariante mediante un modelo de regresión logística de intercepción aleatoria para comparar el grupo control (recordatorio SMS de entrega de TSOH) y el grupo control (no intervención). Se incluirá el área básica de salud como una intercepción de efectos aleatorios. Se calcularán las Odds Ratios y los intervalos de confianza del 95%.

<u>Intervención 3: SMS de invitación en participantes sucesivas del cribado de mama</u>

Diseño: Ensayo controlado aleatorizado
Población de estudio: Todas las mujeres de 52-69 años que hayan participado en el programa de cribado de cáncer de mama en la ronda inmediatamente anterior y con un número de teléfono móvil registrado en nuestra aplicación. Anualmente, se invitan unas 80.000 mujeres, la mitad de las cuales ha participado en la ronda anterior. A diferencia del programa de cribado de CCR, la invitación en el programa no se realiza por área básica de salud sino por fecha de nacimiento. La asignación a los grupos de intervención se hará de forma aleatoria considerando el código de identificación personal de la tarjeta sanitaria (CIP).
Las mujeres de las que no disponemos de su número móvil recibirán la carta de invitación pero no entrarán en el estudio.
Variables: La variable dependiente será la participación en el cribado (sí/no) a las 8 semanas de la fecha inicial de invitación al programa. El tipo de invitación (SMS/carta) será la principal variable independiente y se recogerá el cribado previo, la accesibilidad del correo postal y variables sociodemográficas (edad e índice socieconómico-AQUAS), cambio de cita (sí/no).
Análisis de datos: Se realizará un análisis intermedio (*interim analysis*) a los dos meses del inicio del ensayo, y éste se interrumpirá si la participación puntual en el grupo invitado por SMS en comparación con el invitado por carta se reduce en un 3% o más. Mediante modelos de regresión logística se determinará si la invitación por SMS tiene una participación igual o mejor que la invitación por carta.
Las tres intervenciones incorporarán una <u>evaluación de proceso anidada</u>. Se adoptará un **diseño de métodos mixtos** que incluirá un cuestionario (n=100 por intervención) para testar la lectura y comprensión de los mensajes de texto entre los/las participantes; y entrevistas cualitativas individuales para explorar las experiencias de los receptores/as de las intervenciones 1 (n=36 aprox.), 2 (n=36 aprox.) y 3 (n=24 aprox.). La evaluación de proceso permitirá una interpretación más exhaustiva de los efectos detectados, las posibles variaciones entre subgrupos y las posibles estrategias de optimización de las intervenciones. Además, los datos cualitativos se integrarán y analizarán de modo transversal a las tres intervenciones, poniéndolos en relación con los marcos teóricos de cambio de conducta en los que se fundamentan, para mejorar la comprensión actual del papel de la tecnología móvil en la conducta y toma de decisiones de los participantes en los programas poblacionales de cribado de cáncer.

El <u>análisis de coste-efectividad</u> de las tres intervenciones se iniciará con la evaluación de costes para poder construir posteriormente la ratio incremental coste-efectividad. La efectividad se medirá con el incremento de las personas que han participado en el cribado. Para la evaluación del coste se tendrán en

cuenta los siguientes costes sanitarios que se evaluarán directamente en base a los programas poblacionales de cribado: a) Costes de los programas con especial atención a los costes de personal, material fungible y depreciación del inmovilizado material, así como “overheads”. La alternativa del SMS incluirá los costes fijos del envío (personal e infraestructuras); b) Costes del diagnóstico. Incluirá los costes del estudio y evaluación de los casos con una prueba de cribado positiva. No se incluirán costes futuros. La efectividad se medirá a través del impacto en la participación y,si procede, se calculará la proporción de neoplasias avanzadas detectadas por aumento de participación.
Para el análisis coste-efectividad se construirá un árbol de decisión que tendrá en cuenta la utilización del SMS sobre los costes y los resultados. El resultado de este análisis se especificará como una ratio incremental entre costes y efectividad. La perspectiva de este estudio será la del financiador y el período temporal de análisis será de 1 año.

Limitaciones
Entre las limitaciones del estudio, debemos destacar que para poder incorporar el SMS como nueva vía de comunicación la proporción de usuarios con un número válido ha de ser alta. Disponemos del número de teléfono móvil de más del 85% de la población elegible y esta proporción aumenta año tras año. En los dos estudios previos que realizamos tanto en el cribado de cáncer de mama como en el colorrectal, la proporción de números erróneos era del 9-10%.
El tamaño del SMS puede ser una limitación, ya que debemos limitarnos a 160 caracteres. Sin embargo, podemos añadir un enlace con información más detallada del proceso de cribado. Ejemplo: ICO le recuerda la importancia de prevenir el CCR. Si quiere realizar la prueba acceda a: https://zmz.es/dKmBlg

Aspectos éticos
El Instituto Catalán de Oncología como responsable del tratamiento de los datos de carácter personal de hombres y mujeres de 50 a 69 años del área metropolitana sur de Barcelona invitados a participar en programas poblacionales de detección precoz de cáncer, está procediendo al ajuste y actualización de todos sus protocolos, acuerdos, convenios, cláusulas, etc., al nuevo marco regulador existente en materia de confidencialidad y protección de datos de carácter personal (Reglamento 2016/679 del Parlamento Europeo y del Consejo de 27 de abril de 2016 relativo a la protección de las personas físicas (RGPD) y Ley Orgánica 3/2018, de 5 de diciembre, de Protección de Datos Personales y Garantía de los Derechos Digitales (LOPD-GDD).
La base de datos, con información prospectiva, contará con una variable de identificación codificada, de acceso restringido. El análisis estadístico se realizará sobre información individual pero no permitirá la identificación de los sujetos. El cruce de las diferentes bases de datos estará sometido a secreto estadístico.

Expediente Nº PI/00226

INVESTIGADOR/A PRINCIPAL: MONTSERRAT GARCÍA MARTÍNEZ

MEMORIA DE SOLICITUD PROYECTO DE INVESTIGACIÓN EN SALUD
SECCIÓN PLAN DE TRABAJO

Etapas de desarrollo y distribución de las tareas de todo el equipo investigador, y las asignaciones previstas para el personal técnico que se solicita. Indicar además el lugar/centro de realización del proyecto.

(Ajústese al espacio disponible)

1) **Adaptación de las aplicaciones informáticas de los programas de cribado:** Se prevé la contratación de los servicios externos (empresas informáticas) para que efectúen los cambios necesarios en las aplicaciones informáticas de los programas de cribado que permitan la invitación o recordatorio por SMS. Lugar de realización: empresas externas.

2) **Diseño de las intervenciones del cribado de CCR y presentación a las farmacias colaboradoras del programa:** Todo el equipo investigador participará en la elaboración de los mensajes de texto tanto para el recordatorio de participación como para el recordatorio de entrega de TSOH. Se planificará el circuito y se determinarán qué procedimientos seguir en caso de incidencias con las farmacias colaboradoras. Se realizarán reuniones informativas con las farmacias de cada una de las áreas básicas de salud seleccionadas para el estudio. Lugar de realización: ICO, Colegio Oficial de Farmacéuticos de Barcelona.

3) **Diseño de la intervención en el programa de cáncer de mama:** Todo el equipo investigador contribuirá en la elaboración de los mensajes de texto. Lugar de realización: ICO

4) **Trabajo de campo:** Implementación de las intervenciones. El equipo administrativo de los programas de cribado tendrá un papel importante en la monitorización de incidencias ya que son el primer contacto tanto con los usuarios/as como con las farmacias. Técnico FP contratado (coordinación y monitorización de las intervenciones), Montse García. Lugar de coordinación y monitorización: ICO.

5) **Evaluación de proceso**: Se recogerán datos de proceso de las intervenciones para capturar las perspectivas y experiencias de los/as participantes mediante una estrategia de métodos mixtos. La recogida de datos tendrá lugar en varios momentos temporales desde el envío de los SMS para cada una de las tres intervenciones evaluadas. Albert Farré, Montse García. Lugar de realización: ICO, Universidad de Dundee.

6) **Elaboración y ejecución del plan de análisis:** Una vez finalizado el trabajo de campo, se procederá al análisis de la información. Para cada una de las variables de estudio se aplicarán tests estadísticos adecuados con el fin de estudiar la asociación de estas variables de interés y el efecto en participación. En esta parte del proyecto participarán Núria Milà, Noémie Travier y Montse Garcia. Lugar de realización: ICO

7) **Análisis de coste-efectividad:** Se recogerán los costes sanitarios directos de los programas de cribado. Se realizará una búsqueda de la literatura para obtener los costes no sanitarios. MªJosé Pérez, Montse García. Lugar de realización: ICO

8) **Interpretación, discusión y diseminación de resultados:** Todo el equipo investigador participará activamente en la discusión e interpretación de los resultados. Conjuntamente se elaborará el plan de diseminación de resultados (principales destinatarios: gestores, investigadores, líderes de opinión y población general). El plan incluirá presentaciones en prensa, presentaciones a congresos, publicaciones científicas, informes a la administración y mass media...). Lugar de realización: ICO.

Expediente Nº PI/00226

INVESTIGADOR/A PRINCIPAL: **MONTSERRAT GARCÍA MARTÍNEZ**

MEMORIA DE SOLICITUD PROYECTO DE INVESTIGACIÓN EN SALUD
SECCIÓN PLAN DE TRABAJO
(Ajústese al espacio disponible. Puede incorporar hasta un máximo de 8 líneas de Actividad/Tarea)

CRONOGRAMA

ACTIVIDAD / TAREA	PERSONA/S INVOLUCRADAS		E	F	M	A	M	J	J	A	S	O	N	D
Adaptación de las aplicaciones informáticas de los programas de cribado	Empresas informáticas	1º Año	✓	✓	✓	✓	✓	✓	✓					
		2º Año												
		3º Año												
Diseño de las intervenciones del cribado de CCR y presentación a las farmacias colaboradoras del programa.	Equipo investigador	1º Año				✓	✓	✓			✓	✓	✓	✓
		2º Año												
		3º Año												
Diseño de la intervención en el programa de cáncer de mama	Equipo investigador	1º Año						✓	✓	✓				
		2º Año												
		3º Año												
Trabajo de campo (implementación de las tres intervenciones)	Técnico FP, Montse García (IP)	1º Año												
		2º Año	✓	✓	✓	✓	✓	✓	✓	✓	✓	✓	✓	✓
		3º Año												
Evaluación de proceso	Albert Farré,Montse García (IP)	1º Año												
		2º Año			✓	✓			✓			✓		
		3º Año	✓	✓	✓									
Elaboración y ejecución del plan de análisis	Núria Milà, Noémie Travier, Montse García (IP)	1º Año												
		2º Año							✓	✓	✓	✓		
		3º Año	✓	✓	✓									
Análisis de coste-efectividad	MªJosé Pérez, Montse García (IP)	1º Año												
		2º Año												
		3º Año		✓	✓	✓	✓							
Interpretación, discusión y diseminación de resultados	Equipo investigador	1º Año												
		2º Año												
		3º Año				✓	✓	✓	✓	✓	✓	✓	✓	✓

Expediente Nº
PI/00226

INVESTIGADOR/A PRINCIPAL: **MONTSERRAT GARCÍA MARTÍNEZ**

MEMORIA DE SOLICITUD PROYECTO DE INVESTIGACIÓN EN SALUD
SECCIÓN PLAN DE TRABAJO

Inserte (si lo desea) una imagen con un cronograma. **Máximo un fichero de imagen formato jpg**

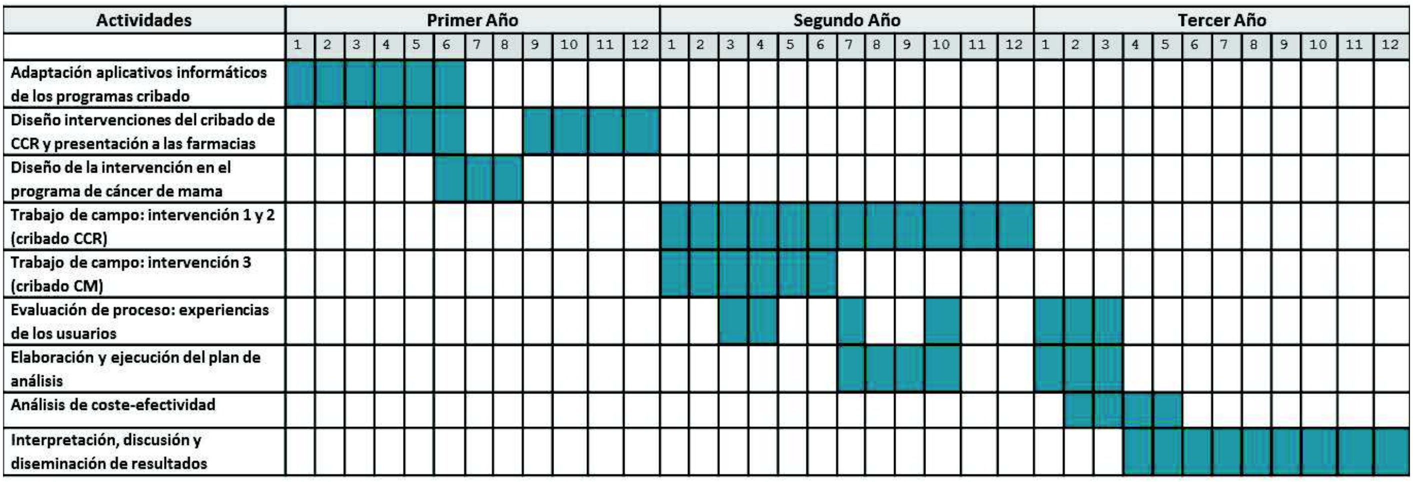

Supplement: S2 File — (PDF) [file pone.0245806.s004.pdf]
